# The Differences in the Level of Anti-SARS-CoV-2 Antibodies after mRNA Vaccine between Convalescent and Non-Previously Infected People Disappear after the Second Dose—Study in Healthcare Workers Group in Poland

**DOI:** 10.3390/vaccines9121402

**Published:** 2021-11-27

**Authors:** Joanna Kwiecińska-Piróg, Jana Przekwas, Zuzanna Kraszewska, Alicja Sękowska, Sylwia Brodzka, Natalia Wiktorczyk-Kapischke, Katarzyna Grudlewska-Buda, Ewa Wałecka-Zacharska, Maciej Zacharski, Aneta Mańkowska-Cyl, Eugenia Gospodarek-Komkowska, Krzysztof Skowron

**Affiliations:** 1 Department of Microbiology, Nicolaus Copernicus University in Toruń, L. Rydygier Collegium Medicum in Bydgoszcz, 85-094 Bydgoszcz, Poland; j.kwiecinska@cm.umk.pl (J.K.-P.); jana.przekwas@doktorant.umk.pl (J.P.); z.kraszewska@cm.umk.pl (Z.K.); asekowska@cm.umk.pl (A.S.); n.wiktorczyk@cm.umk.pl (N.W.-K.); katinkag@gazeta.pl (K.G.-B.); gospodareke@cm.umk.pl (E.G.-K.); 2 Department of Biotechnology, Faculty of Biological Science, University of Zielona Gora, 65-417 Zielona Gora, Poland; sylwia_florczak89@wp.pl; 3 Department of Food Hygiene and Consumer Health, Wroclaw University of Environmental and Life Sciences, 50-375 Wroclaw, Poland; ewa.walecka@upwr.edu.pl; 4 Department of Biochemistry and Molecular Biology, Wroclaw University of Environmental and Life Sciences, 50-375 Wrocaw, Poland; maciej.zacharski@upwr.edu.pl; 5 Department of Laboratory Medicine, Nicolaus Copernicus University in Toruń, L. Rydygier Collegium Medicum in Bydgoszcz, 85-094 Bydgoszcz, Poland; aneta.mankowska@cm.umk.pl

**Keywords:** antibodies, SARS-CoV-2, vaccination, immunological response

## Abstract

(1) Background: In many infections, antibodies play a crucial role in controlling infection. In COVID-19, the dynamics of the immune system response to SARS-CoV-2 is not fully understood. (2) Methods: The study was conducted on 120 healthcare workers from Dr. Antoni Jurasz University Hospital No. 1 in Bydgoszcz, between June and December 2020. In all participants, IgA and IgG antibody serum concentrations were measured using the semi-quantitative Anti-SARS-CoV-2 ELISA test (Euroimmun). After vaccination, in January and February 2021, antibody levels were examined using the quantitative IgG Anti-SARS-CoV-2 Quantivac ELISA test (Euroimmun). (3) Results: During the whole study period, the SARS-CoV-2 infection was confirmed in 29 (24.2%) participants. In all infected participants, IgA and IgG antibodies were detectable after infection by semi-quantitative serological tests. Levels of antibodies were higher one month after the first dose in the convalescents than in the non-previously infected participants. In this second group, the level of antibodies increased significantly after the second dose of vaccines compared to the first dose. (4) Conclusions: The level of antibodies after the first dose of vaccine in the convalescents’ group is higher than in the SARS-CoV-2 non-infected group, but the differences disappear after the second vaccination.

## 1. Introduction

The first case of COVID-19 (Coronavirus disease 2019) caused by SARS-CoV-2 (Severe Acute Respiratory Syndrome Coronavirus 2) was notified in December 2019 (Wuhan, China). In March 2020, the World Health Organization (WHO) announced the worldwide pandemic, which has continued up to now [1]. From 31 December 2019 until 17 November 2021, 255,253,048 cases of COVID-19, including 5,133,226 deaths (in accordance with the applied case definitions and testing strategies in the affected countries), have been reported in 217 countries and territories [2]. The situation is dynamically evolving, with more cases expected in the coming days.

SARS-CoV-2 is complemented by about 14 open reading frames (ORF), encoding structural and non-structural proteins. The SARS-CoV-2 genome encodes approximately 25 proteins, including four major structural proteins: spike (S), envelope (E), membrane (M), and nucleocapsid (N), and 16 non-structural proteins (nsp1-16) [3]. The S protein consists of two subunits, S1 (consists of N-terminal domain (NTD) and receptor-binding domain (RBD), binding the cellular receptor) and S2 (allows the fusion peptide to insert into the host target cell membrane). The N protein binds and packs the viral RNA into a helical structure during the replication stage. In turn, the E protein is involved in the morphogenesis and assembly of viral filaments [4]. SARS-CoV-2 gains entry to human cells by binding the angiotensin-converting enzyme 2 (ACE2) receptor with the RBD of its spike (S) protein [1].

Neutralizing antibodies (nAbs) play a crucial role in controlling viral infection. Earlier studies [5,6] suggest that the S and N proteins of SARS-CoV-2 trigger the production of host neutralizing antibodies. However, the key target of nAbs in SARS-CoV-2 is S protein. nAbs have been detected in symptomatic COVID-19 patients, and their potency seems to be associated with high levels of antibodies [7]. Immunoglobulin G (IgG) targeting the N protein is also detectable in the serum of infected patients [8]. In contrast, IgA antibodies bind to the SARS-CoV-2 RBD [9,10]. Microarray analyses have also revealed antibody responses to other SARS-CoV-2 proteins (mainly ORF9b and nsp5) [11]. Convalescent plasma is applied to treat patients with severe COVID-19. The Food and Drug Administration (FDA) has issued a recommendation that the nAbs titer in the plasma of convalescents should be at least 160, but a titer of 80 is also acceptable [12]. However, this document does not specify the level of SARS-CoV-2 neutralization to be achieved or how to measure it. Researchers [7] have shown that approximately 30% of COVID-19 convalescents (tested two weeks after discharge from hospital) had low neutralizing antibody titers.

**Figure 1:**
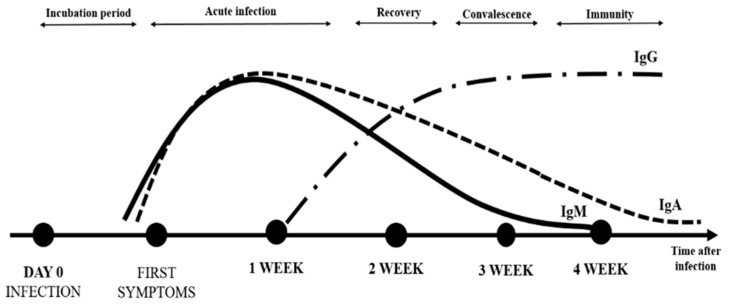
The dynamics of the immune system response to SARS-CoV-2.

The dynamics of the immune system response to SARS-CoV-2 is not fully understood [13]. IgA antibodies appear rapidly (median time of detection in patients is two days after SARS-CoV-2 infection) [3] and can maintain an elevated plasma level for at least 40 days after symptom onset [9,10]. In contrast, the median time of appearance is five- and fourteen-days post-infection for anti-S antibodies (IgM) and IgG antibodies, respectively [7]. The difference in neutralizing activity between IgA and IgG in serum may be due to differences in developmental kinetics during the immune response to SARS-CoV-2 [4,14]. The level of specific antibodies increases rapidly during the first two weeks [1]. In addition, anti-SARS-CoV-2 specific IgG antibodies persist longer (detected for at least 49 days after the onset of symptoms) at a high level, which may suggest their protective role (long-term immunity and immune memory) [7] (Figure 1).

Grzelak et al. [7] have shown that anti-SARS-CoV-2 antibody titers correlate with the course of infection (mild or severe) and possibly with higher rates of viral replication and/or immune activation in severe COVID-19 patients. There was no correlation between age or gender and the intensity/duration of the immune response. In addition, Yu et al. [3] have found significantly higher levels of IgM and IgG in severe COVID-19 patients than those with mild or moderate disease.

Monitoring the level of specific IgG, IgM, and IgA antibodies in the plasma can be a simple way to monitor the immune response in convalescents or after vaccination [15]. The FDA believes that there is a need for more research on vaccinated people. Determination of the presence and dynamics of anti-SARS-CoV-2 antibodies may also serve as a valuable diagnostic tool in people without previous genetic/antigen tests and with complications after COVID-19 [12]. According to the WHO, serological tests are a reliable method to confirm infection in this case.

Due to contact with infected (symptomatic and asymptomatic) patients or colleagues, health care workers are at greater risk of contracting infection than the general population. The seroprevalence of SARS-CoV-2 IgG antibodies among health care workers varies widely by country and region [16,17]. Moreover, the time point of the pandemic (number of infected people in the population) and the department (specialization and seniority) influence the seroprevalence. Cook et al. [18] have reported lower seroprevalence and lower mortality among Intensive Care Units (ICU) staff and anesthesiologists. Conversely, due to more frequent contact with patients, there was a greater likelihood of contracting COVID-19 in the group of acute and internal medicine workers and younger hospital staff [19,20].

Martin et al. [20] demonstrated that ethnicity is also associated with seropositivity for anti-SARS-CoV-2 IgG. Compared to white race workers (9.1% seroprevalence), seroprevalence was higher in South Asian (12.3%) and black race (21.2%) health care workers [20]. Lumley et al. [21] have shown that the mean estimated half-life of IgG antibodies in health care workers was 85 days. Similarly, data from 1215 individuals in Iceland revealed that IgG responses to nucleocapsid and the S1 component of spike were detectable for 100–125 days [22]. However, Robbiani et al. [23] and Muecksch et al. [24] have noted declines in neutralizing antibodies over a similar period. SARS-CoV-2 anti-nucleocapsid antibodies disappear within few months, faster in young adults and asymptomatic individuals, while anti-spike IgGs remain detectable [21].

Healthcare workers are one of the first groups vaccinated against SARS-CoV-2. Therefore, the first data on the dynamics of vaccine-induced antibodies derive from the analysis in this group. In a study by Saadat et al. [25], healthcare workers with previously laboratory-confirmed COVID-19 had higher antibody titers after a single dose of mRNA vaccine than undiagnosed workers. Antibody titers started to peak after seven days and achieved higher titers and neutralization within 14 days compared to COVID-19 negative volunteers [25].

As the COVID-19 pandemic is rapidly expanding, we need to proactively explore plausible options to slow its seemingly inexorable progress and be better prepared for new waves of severe epidemic respiratory infections. Though direct evidence of a link between vitamin D levels and COVID-19 incidence or outcomes is lacking, indirect evidence of an immunomodulatory role of vitamin D in respiratory infections exists, if only by the fact that it has suppressive effects on several inflammatory cytokines centrally implicated in fulminant COVID-19 illness, including interleukin-6 (IL-6), tumor necrosis factor-alpha (TNF-α), and interferon-gamma (IFNγ). Therefore, correcting deficient vitamin D status may be a good target for such an intervention because the possibly seasonal outbreaks of COVID-19 and the previous severe acute respiratory syndrome (SARS) epidemic could point in this direction [26,27].

The main goal of the study was to determine the dynamics of IgG level in healthcare workers after COVID-19 and after vaccination, as well as to determine the usefulness of serological methods in monitoring survivors of the infection. In addition, we decided to check whether the vitamin D supplementation declared by the respondents was related to COVID-19 infection and the immune response.

## 2. Materials and Methods

### 2.1. Methods

BD Vacutainer CAT tubes (Becton-Dickinson) were used for the sample collection. Blood was allowed to clot for 30 min at room temperature and transferred to the Microbiology Clinic during this time. Samples were centrifuged 2500× *g* for 10 min according to the ELISA manufacturer’s instruction. Next, serum was aliquoted into two tubes: one stored at 4 °C until examination (no longer than three days), and the second frozen at −80 °C.

IgA and IgG antibodies serum concentrations were measured using the semi-quantitative Anti-SARS-CoV-2 ELISA test (Euroimmun). During the early stage of our study, no control serum samples for SARS-CoV-2 antibodies were available. Therefore, we used a semi-quantitative test, and the results were interpreted in the ratio of serum absorbance to the cut-off value, as defined by the test manufacturer. For the reason of lack of reliable tests for IgM in the early stage of study, we did not examine the levels of this antibodies class.

In the second stage of the study, in 50 participants after vaccination, antibody levels were examined using the quantitative IgG Anti-SARS-CoV-2 Quantivac ELISA test (Euroimmun). Results were presented as Binding Antibody Units (BAU/mL).

Both assays were done according to the manufacturer’s instructions. Every month, all participants filled out a questionnaire (Supplementary Table S1).

#### 2.1.1. Vitamin D Analysis

The concentration of vitamin D in the examined serum was analysed by using Liaison^®^ 25OH Vitamin D TOTAL Assay test (DiaSorin^®^, Italy) according to the manufacturer instructions. Results were interpreted according to the instructions:

- Deficiency: less than 10 ng/mL
- Insufficient level: 10–30 ng/mL
- Sufficient level: 30–100 ng/mL
- Toxic: more than 100 ng/mL

#### 2.1.2. Statistical Analysis

Statistical analysis was performed using STATISTICA 13 (TIBCO Software Inc. (2017), Statistica (data analysis software system), version 13. http://statistica.io). We used the Mann–Whitney U test and Wilcoxon test to compare the IgA and IgG class antibody levels, and chi-square tests for the differentiation between strong and moderate immune response groups. Excel 2013 (Microsoft) was used to calculate the polynomial regression model for each patient’s antibody level dataset. The coefficient of determination (R^2^) was calculated to evaluate the goodness of fit. In all statistical analyses, the significance level was set at α = 0.05.

## 3. Results

### 3.1. Characteristics of the Study Group

The study was conducted on 120 healthcare workers from Dr. Antoni Jurasz University Hospital No. 1 in Bydgoszcz, which admitted the COVID-19 patients. Table 1 presents the characteristics of the group by gender and age. The volunteers who had contact with patients (104; 86.7%) or with specimens collected from SARS-CoV-2 positive patients (16; 13.3%) were included in the initial study group. The samples from volunteers were collected between June and December 2020 in one-month intervals.

Fifty participants from the above-mentioned initial study group were enrolled in the second study group based on the completeness of the collected specimens and data for the period between July–December 2020. Characteristics of the second study group are presented in Table 2. Participants were divided into two subgroups based on the presence of previous SARS-CoV-infection:

- Convalescents—volunteers who had IgG-positive results at any point of described above period and confirmed SARS-CoV-2 infection by using molecular tests (information from participants on the base of the questionnaires); (17 participants);
- Non-previously SARS-CoV-2 infected people—the control group was IgG-negative throughout the entire period of study, and no information about SARS-CoV-2 infection in questionnaires; (33 participants).

Samples from second study group were collected in January and February 2021 in one-month intervals.

### 3.2. Serological Surveillance Results in the Initial Period: June–December 2020

In July 2020, in the antibody examination of the 120 participants in the initial study group, no people with anti-SARS-CoV-2 IgG were found. Anti-SARS-CoV-2 IgA was found in seven (5.8%) subjects, but these results were interpreted as a cross-reaction. The measurements of their samples were repeated and showed they had at least two IgA-positive (or borderline), while having no IgG antibodies. In the survey, these participants declared allergies, autoimmune or chronic diseases, and almost all of them (*n* = 6, 85.7% cross-reactive samples) had pets at home (Table 3).

During the whole first study period (from July until December 2020), the SARS-CoV-2 infection was confirmed in 29 (24.2%) participants by real-time RT-PCR tests (data not shown). In all infected participants (except one—infected on 31 December 2020, and not examined directly before vaccination—participant No. 71), IgA and IgG antibodies were detectable after infection by semi-quantitative serological tests (Supplementary Table S2). This group was named convalescents. In the rest of the participants (91, 75.8%), named the non-previously infected or control group, neither SARS-CoV-2 RNA was detected, nor were antibodies against SARS-CoV-2 found.

Amongst all 120 participants, 57 (47.5%) declared close contact with SARS-CoV-2 positive patients, but only 3 (2.5%) of them had the positive result of the RT-PCR SARS-CoV-2 test, at most three days after mentioned contact.

The distribution of the number of COVID-19 cases in the following six months in the first study group and its comparison with the distribution of the number of cases in Poland is presented in Table 4. In July, no positive cases of SARS-CoV-2 infection were detected in the study group, but their number in the following months increased, except in September (Table 4). The highest disproportion between the number of cases in the Polish population (0.7%) and the study group (12.5%) was observed in October. Summing up, the percentage of cases in the study group, apart from July, is higher each month than in the general Polish population, as shown in Table 4. The reason may be that the hospital personnel are particularly exposed to contact with SARS-CoV-2.

### 3.3. Pre-Vaccine Antibodies Analysis

**Figure 2:**
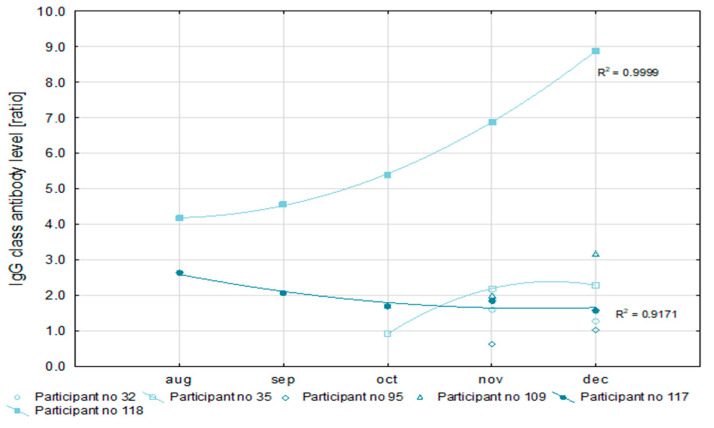
The dynamics of IgG class antibody level in COVID-19 convalescents between July and December 2020 (semiquantitative ELISA assay, Euroimmun) (*n* = 6).

**Figure 3:**
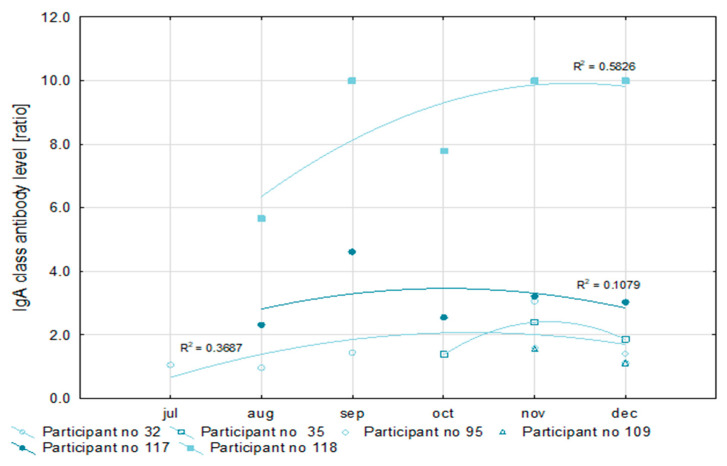
The dynamics of IgA class antibody level in COVID-19 convalescents between July and December 2020 (semiquantitative ELISA assay, Euroimmun) (*n* = 6).

In 20 (68.9%) of the 29 convalescents, SARS-CoV-2 infections were detected in October and November (Table 4). That made a reliable analysis of antibody levels impossible. For this reason, only six patients were qualified for the detailed analysis of the level of antibodies. All convalescents had IgG antibody levels above a 1.1 ratio, which is the cut-off point for a positive result in the semi-quantitative Euroimmun test (Figure 2). At the same time, in half of the examined convalescents, the level of IgG antibodies increased, and in the remainder, it decreased during recovery. The level of IgA antibodies after recovery increased in one of the convalescents, decreased in another, and fluctuated in the remaining four as is shown in Figure 3. However, in all cases, it exceeded the ratio value of 1.1, i.e., the positive cut-off point for the semi-quantitative Euroimmun test.

In two participants, antibody levels could be monitored within five months after the first SARS-CoV-2 detection. Values of the coefficient of determination (R^2^) for the nonlinear regression of anti-SARS-CoV-2 IgG antibodies concentration higher than 0.9 prove the usefulness of this measurement for the assessment of the immune response. Similarly, a low R^2^ value for the IgA antibody concentration (not higher than 0.6) may indicate the poor efficiency of this factor for immune response analysis.

### 3.4. Vitamin D Analysis Results

The sufficient level of vitamin D was obtained in only six (12.0%) of the fifty included in the second study group participants. Most of them supplemented their diet (12; 75.0%). Vitamin D concentration was insufficient in most volunteers (32; 64.0%), even though half of them supplemented their diet (16; 50%). In two cases (4.0%), in volunteers who has not declared diet supplementation, the level was below 10 ng/mL.

### 3.5. Survey Results

In 17 out of the 50 participants of the second study group (34.0%), COVID-19 was confirmed between June and December 2020. Amongst seventeen participants, four (23.5%) declared the presence of an autoimmune disease, seven (41.2%) declared chronic disease, and ten (58.8%) reported having an animal at home. While only eight of the SARS-CoV-2 positive participants (47.1%) supplemented with vitamin D, we found that most (22; 66.7%) of the participants without SARS-CoV-2 infection (non-previously infected group) used vitamin D supplementation; however, the difference was not statistically significant (0.1800; Table 5). The concentration of vitamin D in serum was insignificantly lower in convalescents than in non-previously infected participants (respectively: 24.9 ng/mL and 26.72 ng/mL; results of the Mann–Whitney U test: 0.4010).

### 3.6. Analysis of IgA and IgG Class Antibodies Levels in Serum One Month after the First Dose of the mRNA Anti-SARS-CoV-2 Vaccine (Pfizer-BioNTech)

**Figure 4:**
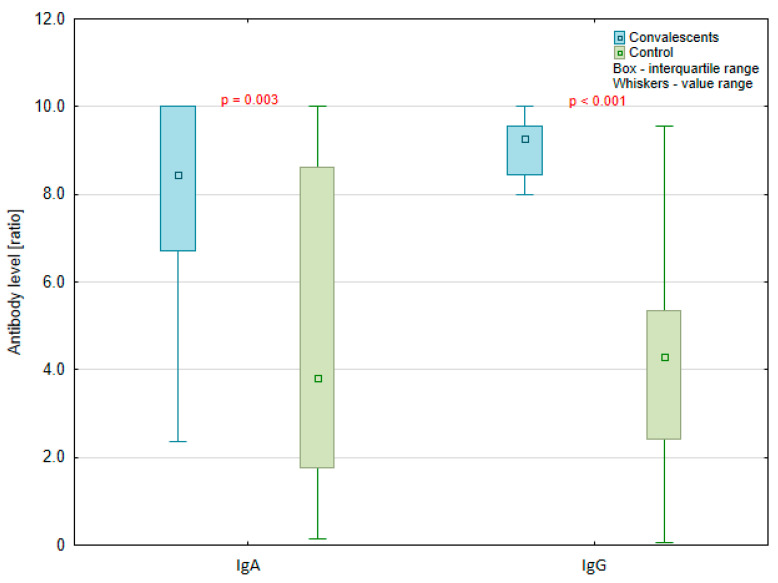
Level of antibodies in IgA and IgG classes one month after the first dose of Pfizer-BioNTech anti-SARS-CoV-2 vaccine expressed in ratio (semi-quantitative Euroimmun test) in the non-previously SARS-CoV-2 infected participants control group (*n* = 33) and in convalescents (*n* = 17).

All participants from the second study group (17 convalescents and 33 negative for SARS-CoV-2 infection) received the Pfizer-BioNTech COVID-19 vaccine (Pfizer Inc., Brooklyn, New York, NY, USA) between the 27th December 2020 and the end of February 2021. Levels of both examined antibody classes were higher one month after the first dose in the convalescents (8.117 mean ratio for class IgA and 8.515 for IgG class) than in the non-previously SARS-CoV-2 infected group (4.747 and 4.299, respectively), and this difference is statistically significant (Figure 4).

### 3.7. IgG Antibodies Analysis after First and Second Dose of the COVID-19 Vaccine (Pfizer-BioNTech)

In the second study group, about 30 days after the first and second dose of vaccine, the concentrations of IgG were examined using a quantitative test. The values of the concentration of IgG antibodies expressed in binding antibody units per 1 mL (BAU/mL) and the absorbance one month after the first and second dose of the vaccine are presented in Table 6. The level of IgG antibodies after the second dose was significantly higher than after the first dose (*p* = 0.000004).

Table 7 shows the median value of the concentration of IgG antibodies (BAU/mL), divided into the groups of convalescents and the non-previously SARS-CoV-2 infected. In SARS-CoV-2 negative participants, as expected, the level of antibodies increased significantly after the second dose of vaccines compared to the first dose. In contrast, COVID-19 convalescents did not show a statistically significant difference in antibody levels between the first and second vaccine doses.

### 3.8. Moderate and a Strong Immune Response

In all healthcare workers, the vaccination immune response was evidenced by increased antibody levels in both IgA and IgG classes. However, even though the level of IgA and IgG antibodies in all studied cases significantly exceeded the cut-off value for the positive result (above a 1:1 ratio in the semi-quantitative Euroimmun test and 35.2 BAU/mL in the quantitative test), it was possible to distinguish participants with moderate and strong immune responses. The cut-off value for these groups was the median level of IgG antibodies after the first dose of the vaccine. The groups are therefore equally numerous (25 cases in each of them). Table 8 presents details of the survey responses of both groups.

The strong immune response was more common in volunteers younger than 50 years old (19, 76.0%) than in people over 50 years old (6, 24.0%), but the difference did not reach a statistical significance.

We did not observe any relationship between vitamin D concentration in serum and the level immune response category (Mann–Whitney U test result: 0.9381). The concentration of vitamin D in the serum of participants with a strong immune response was slightly lower (26.08 ng/mL) than in participants in the moderate category (26.18 ng/mL).

In the group of seven participants with cross-reactions, three had a strong immune response after the first dose of the vaccine. One participant showed a moderate immune response. Unfortunately, the three remaining participants did not enroll in the second stage of the study, and there is no data about the immune response after vaccination.

## 4. Discussion

During the current SARS-CoV-2 pandemic, it has become clear that the magnitude of serological immune response is highly variable [23]. The antibodies specific to SARS-CoV-2 antigens can be detected in most infected individuals 10–15 days after the onset of COVID-19 symptoms. However, due to the recent appearance of this virus in the human population, it is not yet known how long the antibodies will last or whether they will provide protection against reinfection and new virus variants. Serological tests allow the assessment of changes in antibody titers in people diagnosed with COVID-19 disease, as well as in vaccinated people.

Healthcare workers are a professional group that is exceptionally exposed to contact with people infected with SARS-CoV-2 [23]. Many external factors, such as the time and proximity of contact with an infected person, and internal factors, e.g., the functioning of our immune system, affect the probability of infection. The latter is modulated by vitamin D. In our study, people in the control group (without SARS-CoV-2 infection) were more likely to supplement with vitamin D than people who were SARS-CoV-2 positive. These results are consistent with those obtained by other authors [28,29,30]. However, the role of therapeutic vitamin D supplementation in asymptomatic individuals with vitamin D deficiency and SARS-CoV-2 infection is unknown and requires more research. We also found no relationship between immune response and concentration of vitamin D in serum.

During the study, we observed cross-reaction in IgA, mostly in people who have had pets at home (in six of the seven observed cross-reaction cases). Pets at home have 66.7% non-previously SARS-CoV-2 infected and 58.8% convalescents. Empirical observations (Spanish veterinarians) show that the presence of pets may have a positive effect on the course of COVID-19 [31]. However, the correlation between pet ownership and the presence of a mild course of COVID-19 has not yet been demonstrated. The data show [32,33] that the prevalence of canine respiratory coronavirus (CrCoV) in dogs is high, which may suggest that people who own a pet may be more likely to have contact with different types of canine coronaviruses. Jurgiel et al. [34] suggested that re-exposure to animal coronaviruses could lead to immune system stimulation, thus creating an effective response to SARS-CoV-2 infection (immunization). There is a need for more research into the correlation between pet possession and the course of COVID-19.

One of the goals of our study was to evaluate the level of antibodies in healthcare workers positive for SARS-CoV-2. In 50% of the examined convalescents, the level of IgG antibodies increased, and in the remainder it decreased during the study (five months). IgA antibody level changes over time showed high variability between the individuals and have limited predictive value. Studies suggest that the humoral immune response provides primary protection against SARS-CoV-2. Most people experience seroconversion after contracting COVID-19 [23,35]. Shields et al. [36] found that people with the symptomatic disease had significantly higher seroconversion rates than asymptomatic ones. Some studies revealed a lack of seroconversion or even loss of antibodies after infection [37,38]. Patel et al. [39] found that 42 and 58% of health workers in Tennessee (United States) showed a sustained IgG response and a labile IgG response, respectively, 60 days after diagnosis. Robbiani et al. [23] demonstrated that COVID-19 patients recovering from an infection did not have high levels of neutralizing antibodies 39 days after the onset of symptoms. Demonbreun et al. [40] found a loss of seropositivity of anti-SARS-CoV-2 antibodies after 120 days in 25% of people tested in Chicago (United States). Bichara et al. [41] also showed a high frequency of loss of anti-SARS-CoV-2 IgG antibodies within three months after COVID-19 diagnosis in the Brazilian Amazon. These results indicate that some people do not develop a measurable immune response after contracting COVID-19.

So far, we are not able to answer the question: what level of antibodies guarantees an immune response that protects both convalescents and those vaccinated against reinfection? Analyzing the antibody levels of six convalescents, we noted that all participants after COVID-19 had IgG antibody levels above 1.1, which is the cut-off point for a positive result in the semi-quantitative Euroimmun test. Variations in IgG and IgA antibody levels may result from the continued exposure of post-illness healthcare workers to SARS-CoV-2 positive patients and infectious material. We monitored the antibody levels of two participants within five months after they were first diagnosed with SARS-CoV-2. R^2^ coefficient values higher than 0.9 for the concentration of anti-SARS-CoV-2 IgG antibodies prove the usefulness of this measurement for the immune response assessment. Conversely, a low R^2^ value for the level of IgA antibodies (not higher than 0.6) may indicate poor efficiency of this factor for the immune response analysis. This is consistent with the results of other authors who consider the level of IgG antibodies to be a useful indicator of the actual immune response to SARS-CoV-2 infection [38,41].

The essential element of the presented research was the determination of antibody levels in vaccinated people. Our study included convalescents and non-previously SARS-CoV-2 infected participants vaccinated with the Pfizer-BioNTech vaccine. In both examined groups, the level of IgG antibodies was higher after the second dose of the vaccine than after the first dose. The immune response level after the first vaccination was statistically significantly different in these groups. The titer of IgG antibodies was higher in convalescents (1969.9 BAU/mL) than in non-previously SARS-CoV-2 infected participants (717.5 BAU/mL). Krammer et al. [42] obtained similar results demonstrating that the majority of seronegative participants had varied and low IgG antibody levels within 9 to 12 days after vaccination; this is related with the repeated exposure of the SARS-CoV-2 antigens in convalescents. In vaccinated people, it is the first contact with the SARS-CoV-2 antigen, so the immune response is usually lower than in the recovering group, whose immune system recognizes the antigen and produces antibodies more effectively. In this study, subjects with SARS-CoV-2 antibodies present before the first vaccination rapidly developed uniform, high antibody titers within days after vaccination. Ebinger et al. [43] observed a similar immune response in both healthy and SARS-CoV-2 convalescents. In our study, the initial differences in the number of antibodies one month after the first dose between both groups disappeared one month after the second dose of the vaccine. Probably, in recovering people, the antibody levels after every next dose of vaccination will be similar to the level obtained after the first vaccination. Non-previously infected people need at least two doses of vaccine to obtain the same antibody levels as recovering people. The combination of COVID-19 recovery and vaccination gives “hybrid immunity” that is probably related to memory B cells that are activated more effectively during infection than after vaccination [44].

People under 50 years produced a strong immune reaction after the first vaccination, more often than the elderly. It is well-known that age-related changes in the immune system affect cells and mediators of immune responses [45]. Yang et al. [46] observed that younger children exhibited higher IgG levels than adolescents and young adults. This suggests that age is a relevant factor in specific antibody responses. While antibody levels are not the only factors influencing the level of immunologic response, they are reliable, easy to measure, and frequently used for analysis. A large cohort study conducted so far indicates that we are currently unable to predict the duration of the immune response in vaccinated individuals that would protect against SARS-CoV-2 infection [47]. Possibly, a third dose of the vaccine will be required to maintain the antibody titer at a level that guarantees safety and prevents reinfection. Long-term monitoring may give additional information on the expected duration of acquired immunity after a single dose compared to a double dose of the vaccine. The main limitations of our study were the short antibody levels monitoring time and the abundance of the study group.

## 5. Conclusions

In conclusion, the level of antibodies after the first dose of vaccine in the convalescents’ group is higher than in the non-previously SARS-CoV-2 infected group, but the differences disappear after the second vaccination. However, much lower IgG levels after the first mRNA vaccine in non-previously SARS-CoV-2 infected people may force the necessity of the third vaccine dose in this subpopulation, especially in the elderly. Although we did not obtain the statistically significant age-dependent differences between the intensity of the immune response, the strong immune response is less prevalent in older than in younger people. It is highly probable that age-related biological changes not only influence the host immune response to the infection, but also the vaccine efficiency.

## Figures and Tables

**Table 1:** Age and gender of the first study group participants (*n* = 120).

Age Range	Number	Percentage (%)
<30	21	17.5
30–39	18	15.0
40–49	37	30.8
50–59	36	30.0
≥60	8	6.7
Gender	Number	Percentage (%)
male	18	15.0
female	102	85.0

**Table 2:** Age and gender of the second study group participants (*n* = 50).

Age Range	Number	Percentage (%)
<30	11	22.0
30–39	5	10.0
40–49	15	30.0
50–59	16	32.0
≥60	3	6.0
Gender	Number	Percentage (%)
male	9	18.0
female	41	32.0

**Table 3:** Survey answers among the participants with immunology cross-reactions (*n* = 7).

Survey	Number of Positive Answers	Percent of Positive Answers (%)
Allergies	2	28.6
Autoimmune diseases	1	14.3
Chronic diseases	2	28.6
Pets at home	6	85.7

**Table 4:** The comparison of the number of new positive SARS-CoV-2 cases in the population of Poland (*n* = 3.8 × 10^7^) and the study group (*n* = 120).

Month-Year	Confirmed COVID-19 Cases in Population of Poland		Confirmed COVID-19 in Study Group	
	*n*	%	*n*	%
Jun-20	10,607	0.03	2	1.66
Jul-20	11,295	0.03	0	0.00
Aug-20	21,684	0.06	1	0.83
Sep-20	24,142	0.06	6	5.00
Oct-20	271,217	0.71	15	12.50
Nov-20	612,420	1.61	5	4.17

**Table 5:** Characteristic of convalescents and non-previously SARS-CoV-2 infected participants (*n* = 50).

Survey Responses		Non-Previously SARS-CoV-2 Infected (*n* = 33)		Convalescents (*n* = 17)		Fisher *p*-Value Result
		Number	Percentage (%)	Number	Percentage (%)	
Age	≥50	14	42.4	5	29.4	0.5398
	<50	19	57.6	12	70.6	
Sex	F	27	81.8	14	82.4	1.000
	M	6	18.2	3	17.6	
Allergies	Yes	3	9.1	0	0	0.5420
	No	30	90.9	17	100.0	
Autoimmune diseases	Yes	5	15.2	4	23.5	0.4675
	No	28	84.8	13	76.5	
Chronic diseases	Yes	15	45.5	7	41.2	1.000
	No	18	54.5	10	58.8	
Pets at home	Yes	22	66.7	10	58.8	0.7568
	No	11	33.3	7	41.2	
Vitamin D supplementation	Yes	22	66.7	8	47.1	0.2293
	No	11	33.3	9	52.9	
Vitamin D concentration	Deficiency	1	3.1	1	5.9	1.000
	Insufficient level	21	63.6	11	64.7	
	Sufficient level	11	33.3	5	29.4	

**Table 6:** The comparison of the concentration of IgG antibodies of all selected participants (*n* = 50) expressed in binding antibody units (BAU/mL) one month after the first and second dose of Pfizer’s SARS-CoV-2 vaccine.

	Median	Min.	Max.	IQR	CV	*p*-Value
1 month after 1st dose	460.8	24.0000	2880.0	2253.6	100.10	0.000004
1 month after 2nd dose	2874.9	103.9230	2880.0	1219.6	38.04	

IQR, interquartile range (IQR); CV, coefficient of variation.

**Table 7:** The median value of IgG antibody concentrations of all selected participants (*n* = 50) expressed in binding antibody units (BAU/mL) one month after the first and second dose of Pfizer’s SARS-CoV-2 vaccine with the distinction of the convalescent and non-previously SARS-CoV-2 infected participants.

	1 Month after 1st Dose		1 Month after 2nd Dose		*p*-Value
	Median	IQR	Median	IQR	
Convalescents	2880.0	2364.78	2880.0	1033.7	0.8791
Non-previously SARS-CoV-2 infected	363.4	503.1	2880.0	1350.0	0.0005

**Table 8:** Summary of the chosen study members (*n* = 50) survey data divided into groups (according to the immune response: strong; moderate).

		Strong Immune Response (*n* = 25)		Moderate Immune Response (*n* = 25)		Fisher *p*-Value Result
		Number	Percentage (%)	Number	Percentage (%)	
Age	≥50	6	24.0	12	48.0	0.139
	<50	19	76.0	13	52.0	
Sex	F	20	80.0	21	84.0	1.000
	M	5	20.0	4	16.0	
Allergies	Yes	1	4.0	1	4.0	1.000
	No	24	96.0	24	96.0	
Autoimmune diseases	Yes	6	24.0	3	12.0	0.464
	No	19	76.0	22	88.0	
Chronic diseases	Yes	9	36.0	13	52.0	0.393
	No	16	64.0	12	48.0	
Pets at home	Yes	14	56.0	18	72.0	0.377
	No	11	44.0	7	28.0	
Vitamin D supplementation	Yes	13	52.0	17	68.0	0.387
	No	12	48.0	8	32.0	
Vitamin D concentration	Deficiency	1	4.0	1	4.0	1.000
	Insufficient level	16	64.0	16	64.0	
	Sufficient level	8	32.0	8	32.0	

## Data Availability

Most data are included in Supplementary Files. More details data are available in authors.

## Supplementary Materials

The following are available online at https://www.mdpi.com/article/10.3390/vaccines9121402/s1, Table S1: Tested person card; Table S2: Results (ratio and its interpretation) of semi-quantitative serological tests in the convalescents group.
