# Supplementary material for: The Differences in the Level of Anti-SARS-CoV-2 Antibodies after mRNA Vaccine between Convalescent and Non-Previously Infected People Disappear after the Second Dose—Study in Healthcare Workers Group in Poland
Source: Vaccines (Basel). 2021 Nov 27;9(12):1402. doi: 10.3390/vaccines9121402 (PMC8707727; doi:10.3390/vaccines9121402)
Supplement: Supplementary file 1 [file vaccines-09-01402-s001.zip › vaccines-1450390-supplementary.pdf]

vaccines MDPI

Supplementary Table S1

TESTED PERSON CARD

First Name and Last Name: ……………………………………...…………………………

Date and place of birth:……………………………………………………………………...

Address: ………………………………………………………………………………….

Contact phone number, e-mail: ………………………………………………………….

Gender: **M** **F**

Test date: ………………………………………………………………………………….

Age (in years): …………………………………………………………………………

Smoking: **YES / NO**

Have you ever been diagnosed with the presence of SARS-CoV-2 RNA? If so, at what time before the test? **YES / NO** ……………….…………………………………………………….

Have you had contact with a person who was diagnosed with SARS-CoV-2 RNA (if so, when)? **YES / NO** …………………………………………………………………..…………..

Have you been in quarantine for COVID-19 (if so, when)? **YES / NO** ……………………….

Have you had a respiratory infection in the last six months? **YES / NO**

If YES:

- when? …………………………. and how long did it take? ……………………………
- what were the symptoms (please underline)?: cough / runny nose / fever / low-grade fever / muscle or joint pain / sore throat / diarrhea / conjunctivitis / headache / loss of taste or smell / skin rash / discoloration of fingers and toes / difficulty breathing or shortness of breath / chest pain or tightness / loss of speech or motor skills / disorientation

Have you traveled outside of your place of residence in the last six months (if so, where - region / country)? **YES / NO** …………………………………………………………………..

Do you have a pet? **YES / NO** (what?) ………………………………………..…

Past infectious diseases in the last 5 years **YES / NO** (what?) ………………………………..
……………………………………………………………………………………...

Chronic diseases **YES / NO** (what?) ……………………………………………………

Autoimmune diseases **YES / NO** (what?) ……………………………………………….

Allergies **YES / NO** ……………………………………………………………………….

Vitamin D supplementation **YES / NO** ……………………………………………

Supplementary Table S2

Patient ID		Date of positive SARS-CoV-2 result	July		August		September		October		November		December/*January	
			IgA	IgG	IgA	IgG	IgA	IgG	IgA	IgG	IgA	IgG	IgA	IgG
1.	117	2020-06-11	n-ex	n-ex	+ (2.300)	+ (2.630)	+ (4.604)	+ (2.054)	+ (2.531)	+ (1.691)	+ (3.218)	+ (1.832)	+ (3.038)	+ (1.576)
2.	118	2020-06-11	n-ex	n-ex	+ (5.664)	+ (4.172)	+ (> 9.99)	+ (4.558)	+ (7.787)	+ (5.397)	+ (> 9.99)	+ (6.878)	+ (> 9.9)	+ (8.895)
3.	109	2020-09-15	-	-	-	-	-	-	-	-	+ (1.569)	+ (1.986)	+ (1.117)	+ (3.182)
4.	32	2020-10-03	+/- (1.036)	-	+/- (0.961)	-	+ (1.428)	-	n-ex	n-ex	+ (3.055)	+ (1.584)	+ (1.108)	+ (1.268)
5.	35	2020-10-05	-	-	-	-	-	-	+ (1.363)	+/- (0.908)	+ (2.389)	+ (2.187)	+ (1.856)	+ (2.279)
6.	122	2021-10-10	n-ex	n-ex	n-ex	n-ex	n-ex	n-ex	-	-	+ (5.415)	+ (6.615)	+ (1/759)	+ (7.135)
7.	95	2020-10-26	-	-	-	-	-	-	-	-	+ (1.574)	- (0.611)	+ (1.403)	+/- (1.011)
8.	70	2020-10-29	-	-	-	-	-	-	-	-	n-ex	n-ex	+ (7.875)	+ (4.234)
9.	116	2020-10-30			-	-	-	-	-	-	n-ex	n-ex	+/- (0.943)	+/- (0.958)
10.	42	2020-11-02	-	-	-	-	-	-	+/- (0.839)	-	n-ex	n-ex	+ (7.183)	+ (5.261)
11.	54	2020-11-04	-	-	n-ex	n-ex	n-ex	n-ex	n-ex	n-ex	n-ex	n-ex	+ (5.420)	+ (5.824)
12.	112	2020-11-05	n-ex	n-ex	-	-	-	-	-	-	-	-	>9.999*	9.381*
13.	56	2020-11-14	-	-	-	-	-	-	-	-	-	-	7.692*	4.976*
14.	9	2020-11-15	-	-	-	-	-	-	-	-	-	-	0.632*	3.330*
15.	28	2020-11-18	-	-	-	-	-	-	-	-	-	-	- (0.667)	+/- (0.926)
16.	76	2020-11-22	-	-	-	-	-	-	-	-	-	-	+ (3.669)	+ (5.160)
17.	53	2020-11-23	-	-	-	-	-	-	-	-	n-ex	n-ex	+ (6.071)	+ (2.482)
18.	89	2020-11-23	-	-	-	-	-	-	-	-	-	-	+ (2.45)	+ (2.558)
19.	83	2020-11-27	-	-	-	-			-	-	n-ex	n-ex	+ (5.748)	+/- (0.921)
20.	86	2020-11-27	-	-	-	-	-	-	-	-	-	-	+ (2.632)	+ (1.973)
21.	50	2020-11-30	-	-	-	-	-	-	-	-	-	-	+ (1.456)	+ (4.876)
22.	85	2020-11-30	-	-	-	-	-	-	-	-	-	-	+ (7.891)	+ (3.445)
23.	87	2020-11-30	-	-	-	-	-	-	-	-	-	-	+ (2.530)	- (0.344)
24.	13	2020-11-30	-	-	-	-	-	-	-	-	-	-	+ (8.023)	+ (2.013)
25.	73	2020-12-01	-	-	-	-	-	-	-	-	-	-	- (0.671)	+ (3.944)
26.	82	2020-12-03	-	-	-	-	-	-	-	-	-	-	2.364*	2.741*
27.	34	2020-12-07	-	-	-	-	n-ex	n-ex	-	-	-	-	6.653*	>9.999*
28.	41	2020-12-07	-	-	-	-	-	-	+/- (0.821)	-	n-ex	n-ex	+ (4.425)	+ (1.705)
29.	71	2020-12-31	-	-	-	-	-	-	-	-	-	-	- / n-ex	- / n-ex

Abbreviation: n-ex – non examined (absence of participants - related with disease or quarantine/isolation); * specimen collected in January 2021; + positive result; - negative result; + /- borderline result; () ratio of antibodies in semi-quantitative test (Euroimmun)
